# Potential Crosstalk between Liver and Extra-liver Organs in Mouse Models of Acute Liver Injury

**DOI:** 10.7150/ijbs.41293

**Published:** 2020-02-10

**Authors:** Yufan Zheng, Baiping Cui, Wenrui Sun, Sining Wang, Xu Huang, Han Gao, Fei Gao, Qian Cheng, Limin Lu, Yanpeng An, Xiaobo Li, Ning Sun

**Affiliations:** 1 Department of Physiology and Pathophysiology, State Key Laboratory of Medical Neurobiology, School of Basic Medical Sciences, Fudan University, Shanghai 200032, China; 2 Department of Pathology, School of Basic Medical Sciences, Shanghai University of Traditional Chinese Medicine, Shanghai 200032, China; 3 State Key Laboratory of Genetic Engineering, School of Life Sciences, Metabolomics and Systems Biology Laboratory, Human Phenome Institute, Fudan University, Shanghai 200433, China; 4 Department of Cardiology, Huashan Hospital, Fudan University, Shanghai 200032, China; 5 Department of Internal Medicine, Huashan Hospital West Campus, Fudan University, Shanghai 200032, China; 6 Shanghai Key Laboratory of Clinical Geriatric Medicine, Fudan University, Shanghai 200032, China

**Keywords:** acute liver injury, systematic change, metabolomics, transcriptomics, crosstalk

## Abstract

Carbon tetrachloride (CCl4), Concanavalin A (ConA), bile duct ligation (BDL), and liver resection (LR) are four types of commonly used mouse models of acute liver injury. However, these four models belong to different types of liver cell damage while their application situations are often confounded. In addition, the systematic changes of multiple extra-liver organs after acute liver injury and the crosstalk between liver and extra-liver organs remain unclear. Here, we aim to map the morphological, metabolomic and transcriptomic changes systematically after acute liver injury and search for the potential crosstalk between the liver and the extra-liver organs. Significant changes of transcriptome were observed in multiple extra-liver organs after different types of acute liver injury despite dramatic morphological damage only occurred in lung tissues of the ConA/BDL models and spleen tissues in the ConA model. Liver transcriptomic changes initiated the serum metabolomic alterations which correlated to transcriptomic variation in lung, kidney, and brain tissues of BDL and LR models. The potential crosstalk might lead to pulmonary damage and development of hepatorenal syndrome (HRS) and hepatic encephalopathy (HE) during liver injury. Serum derived from acute liver injury mice damaged alveolar epithelial cells and human podocytes *in vitro*. Our data indicated that different types of acute liver injury led to different transcriptomic changes within extra-liver organs. Integration of serum metabolomics and transcriptomics from multiple tissues can improve our understanding of acute liver injury and its effect on the other organs.

## Introduction

The liver is the largest solid organ in the body occupying 2.5% of total body weight. It serves vital physiological functions including macronutrient metabolism, detoxification, digestion, hormone regulation, blood volume regulation, and immune system support etc. 1, 2. Various hepatotoxic factors including hepatitis viruses (hepatitis A, B, and E), drugs (especially acetaminophen), immunologic injury, and other factors can cause death of a large number of liver cells, resulting in acute liver injury (ALI) or life-threatening acute liver failure (ALF) 3-5. Hepatorenal syndrome (HRS) and hepatic encephalopathy (HE) are two vital complications of ALF 6, 7. Animal models of acute liver injury provide an excellent tool to understand the pathophysiological mechanisms and to test therapeutic strategies. Commonly used acute liver injury models include chemical injury models (carbon tetrachloride (CCl4), galactosamine, thioacetamide, aflatoxin, etc.), immune injury models (bacillus calmette-guerin vaccine with lipopolysaccharide, concanavalin A (ConA), etc.), cholestatic injury models through bile duct ligation (BDL), and acute liver failure induced by major liver resection (LR).

CCl4 activates cytochromes P-450 (CYP2E1, CYP2B1 or CYP2B2) to form CCl3 radical, leading to reactive oxygen species (ROS) production, lipid peroxidation, and mitochondrial dependent liver injury 8-11. Owing to the increasing incidence of autoimmune hepatitis (AIH) worldwide 12, the model of AIH is widely needed for scientific research. ConA-induced liver injury was considered as an ideal model for AIH 13. As a lectin, ConA binds to mannose residue of glycoprotein and then induces hepatocytes death through stimulating CD4+ T cells, NKT cells and KCs in liver, leading to secretion of pro-inflammatory cytokines and chemokines 11, 14. For cholestasis hepatopathy, BDL is commonly used for rodent models. BDL is a surgically induced extrahepatic biliary obstruction that results in cholestasis, jaundice and hepatocellular damage 15. Although partial hepatectomy (PH) in rodents is a well‐established model to investigate the complex processes involved in liver regeneration 16, massive loss of liver parenchyma in major liver resection model (>75% hepatectomy) will cause severe liver dysfunction and lead to acute liver failure 17, 18.

The limitation of reductionism in current life science studies calls for a more holistic strategy in next generation medical research activities 19. From a holistic view, CCl4 or ConA not only target on liver but also enter the circulation and infiltrate all extra-liver organs, actually producing a systemic toxicity. Although BDL and LR are two liver specific surgical models, extra-liver organs might also be influenced by liver injury. As reported, BDL is a model of intrapulmonary vasodilatation and hepatopulmonary syndrome 20. BDL rats had a reduction in thickness and an increase in the diameter of the artery wall in lung 21. BDL rats also were reported to produce progressive renal dysfunction without structural changes in the kidney, characterizing as HRS 22, 23. ALF induced by LR eventually led to severe complications including HE and multiple organ dysfunction (MODS) 24. Patients after the hepatectomy are at certain risk of kidney damages and even acute kidney failure 25.

There is a lack of investigation summarizing multiple organ changes in these models and exploring the intrinsic molecular mechanism systematically. Omics technology makes possible the systematic study of changes in multiple organs after acute liver injury. In this study, morphological, serum metabolomic and transcriptomic changes of multiple organs (heart, lung, kidney, spleen, brain) were investigated to analyze the systematic changes in the above four commonly used acute liver injury mice models. We found that the transcriptome of multiple extra-liver organs changed significantly. Through analyzing the correlations between organic transcriptome and serum metabolome, we built potential crosstalk networks between liver and serum as well as between serum and extra-liver organs. Our study provided a guidance for selection of the four acute liver injury mice models in research. Furthermore, the present study helps to better understand the influence of acute liver injury on extra-liver organs at the early stage and the potential crosstalk between liver and extra-liver organs.

## Results

### Histological changes of multiple organs in the four mouse models of acute liver injury

**Figure 1:**
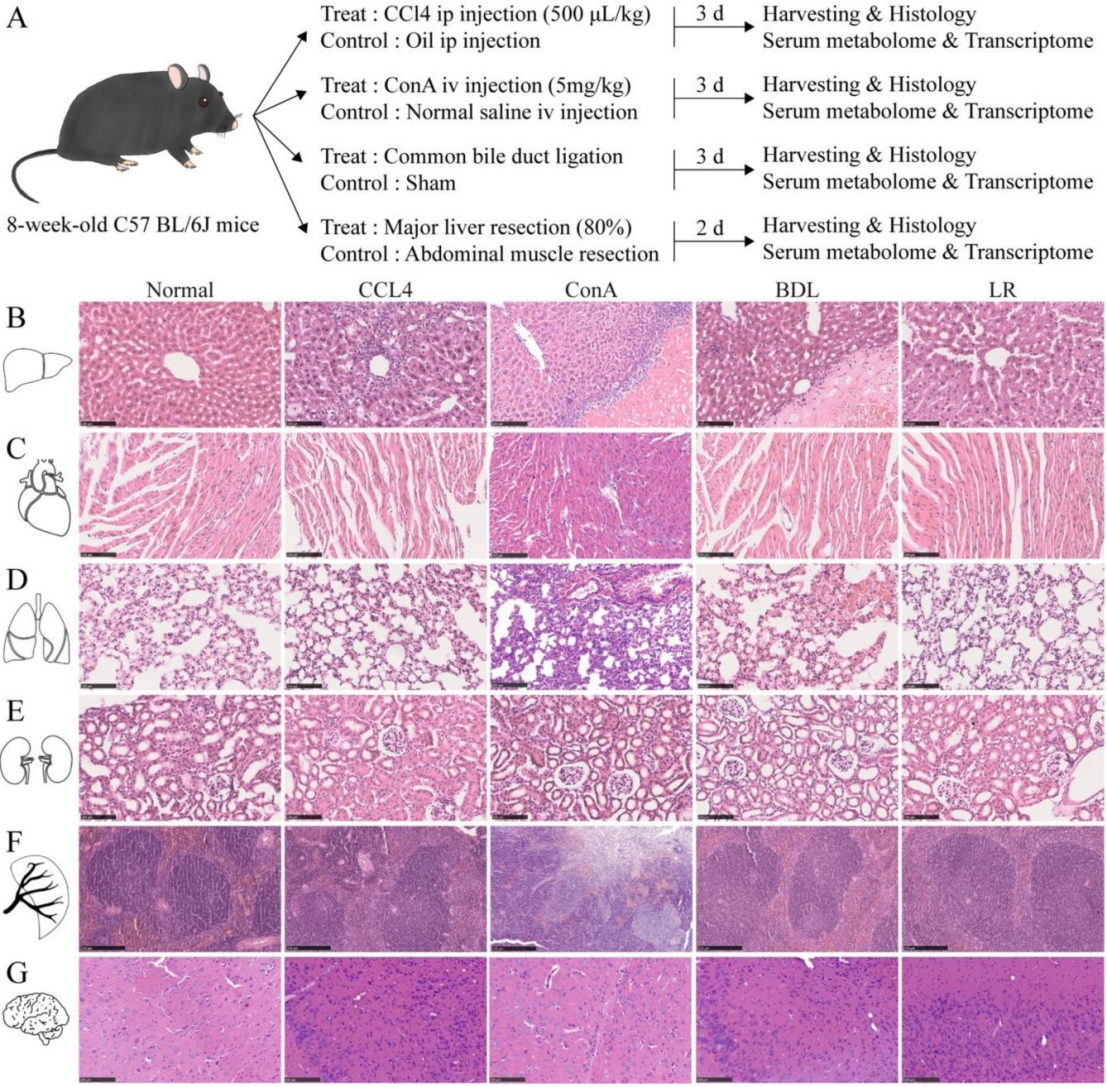
** Experimental design of this study and the representative tissue section images of multiple organs in the 4 mice models of acute liver injury.** (A) Illustration of the experimental design of this study. Liver, heart, lung, kidney, spleen, brain, and serum were collected 3 d after CCl4/oil injection, ConA/NS injection, BDL/Sham surgery, and 2 d after LR/MR. Mice in each treated group and its corresponding control group are from a single cohort of C57BL/6J mice. (B) Representative H&E staining of liver tissue sections from the 4 models of acute liver injury (n=3). Scale bar, 100 μm. (C) Representative H&E staining of heart tissue sections from the 4 models of acute liver injury (n=3). Scale bar, 100 μm. (D) Representative H&E staining of lung tissue sections from the 4 models of acute liver injury (n=3). Scale bar, 100 μm. (E) Representative H&E staining of kidney tissue sections from the 4 models of acute liver injury (n=3). Scale bar, 100 μm. (F) Representative H&E staining of spleen tissue sections from the 4 models of acute liver injury (n=3). Scale bar, 250 μm. (G) Representative H&E staining of brain tissue sections from the 4 models of acute liver injury (n=3). Scale bar, 100 μm. (CCl4, carbon tetrachloride. ConA, concanavalin A. BDL, bile duct ligation. MR, muscle resection. LR, liver resection.)

To see whether acute liver injury affects other organs, liver, heart, lung, kidney, spleen, and brain from the four mouse models of acute liver injury were collected, sectioned, and stained as indicated (**Figure 1A**). H&E staining showed dramatic pathological damage in livers from these four acute liver injury models such as steatosis, inflammatory infiltration, and even hepatocyte necrosis (**Figure 1B**). Perialveolar telangiectasis was observed in the liver tissues from ConA and BDL group (**Figure 1B**) and alveolar congestion was obvious in BDL group (**Figure 1D**). In the spleen tissues from ConA group, the red and white pulps were not well defined and massive necrosis was observed (**Figure 1F**). No significant histological changes were observed in other organ (**Figure 1, C, E, and G**). For the limitation of the space of the main figure, we performed larger view of these section images in **Supplementary Figure 1, 2, 3**.

### Tissue-specific transcriptomic profiling in the four mice models of acute liver injury

**Figure 2:**
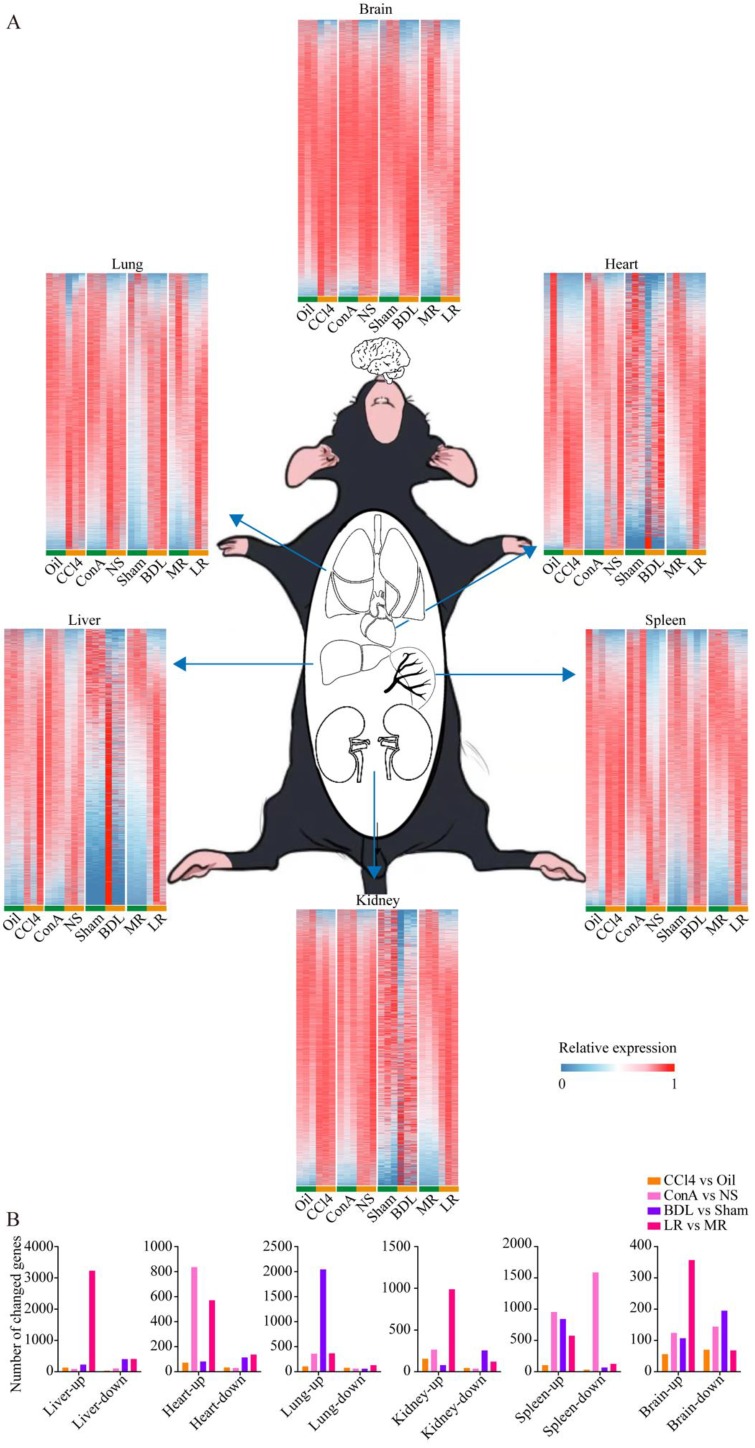
** Tissue-specific transcriptomic profiling in the four mice models of acute liver injury.** (A) Tissue-specific transcriptome heatmaps of the 4 mice models of acute liver injury. Rows reflect normalized relative expression of mRNAs (0-1). (B) The number of genes with significant change in expression (Foldchange>2 and P-value<0.05) in each organ from the 4 models of liver injury. (CCl4, carbon tetrachloride. NS, normal saline. ConA, concanavalin A. BDL, bile duct ligation. MR, muscle resection. LR, liver resection.)

To investigate the changes of multiple organs after acute liver injury at the transcriptional level, large-scale tissue-specific transcriptomic profiling in the four acute liver injury models was performed (**Figure 2A**). Volcano plots showed the number of genes with significant expression changes (**Supplementary Figure 4**). The number of genes with changed expression in each organ was summarized in** Figure 2B**.

We performed Gene Ontology (GO) enrichment for liver transcriptome in four models and found some well-known features of these four models. In CCl4 models, response to chemical, cellular response to chemical stimulus and response to oxygen-containing compound were enriched (**Supplementary Figure 5A**), suggesting CCl4 invoked chemical stimulus and induced oxidative injury in liver. In ConA models, immune system process, immune response and innate immune response were enriched (**Supplementary Figure 5B**), suggesting ConA induced immune injury in liver. In BDL models, regulation of fibroblast proliferation and fibroblast proliferation were enriched (**Supplementary Figure 5C**), suggesting BDL induced liver fibrosis. In LR models, mitotic cell cycle process, cell division and nuclear division were enriched (**Supplementary Figure 5D**), suggesting that liver resection induced liver cell proliferation. These features were well-known features in every model and suggested that our study was reasonable.

Pathway enrichments for each organ were analyzed (**Supplementary Figure 6**), which gave us a systematic insight into the transcriptomic changes of multiple organs when suffered acute liver injury. Venn plots showed co-upregulated and co-downregulated genes in the same organ of four models (**Supplementary Figure 7**). No genes were co-upregulated or co-downregulated in all four models, and there were several genes co-upregulated in three models in lung, kidney, and spleen tissues as indicated.

### The serum NMR metabolome in the four acute liver injury mice models

**Figure 3:**
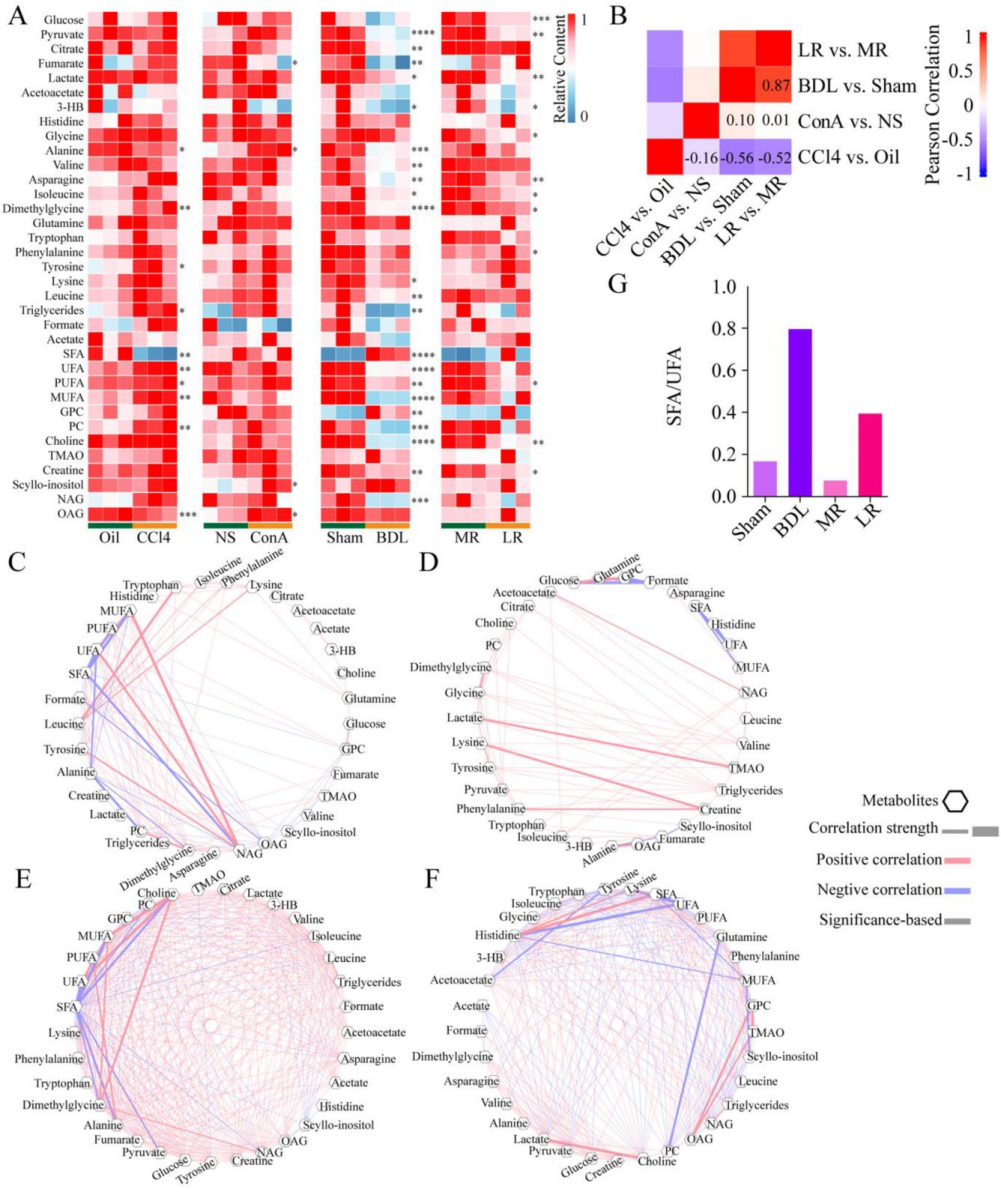
** The serum NMR metabolome in the four acute liver injury mice models.** (A) Serum metabolome heatmaps of the 4 models of acute liver injury. Significance was indicated on the right side of each heatmap. (B) Correlation heatmap between the 4 models. Correlation coefficient rho is shown as red (positive) or blue (negative). Pearson coefficient is marked in the figure. Correlations between metabolites in CCl4 (C), ConA (D), BDL (E), and LR (F) models are shown. Pearson coefficient with an absolute value greater than 0.75 was displayed. Each correlation was shown as a line across metabolites. The width of the line indicated the correlation strength. Positive correlation was shown as red lines and negative correlation as blue lines. (G) The ratio of SFA/UFA in BDL/Sham and LR/MR models. *P < 0.05, **P<0.01, ***P<0.001, ****P<0.0001 by unpaired T test (Two-sided). (CCl4, carbon tetrachloride. NS, normal saline. ConA, concanavalin A. BDL, bile duct ligation. MR, muscle resection. LR, liver resection. 3-HB, 3-hydroxybutytrate. SFA, saturated fatty acid. UFA, unsaturated fatty acid. MUFA, monounsaturated fatty acid. PUFA, polyunsaturated fatty acid. GPC, glycerophosphorylcholine. PC, phosphorylcholine. TMAO, trimetlylamine oxide. NAG, N-acetylated glycoproteins. OAG, O-acetylated glycoproteins.)

Blood from mice was collected as indicated in **Figure 1A** and metabolite profiling of serum was performed (**Figure 3A**). A total of 35 metabolites was detected. Partial least squares discrimination analysis (PLS-DA) revealed a good distinction between metabolites content in treated group and that in control group (**Supplementary Figure 8**).

The changes of metabolite profiling showed different patterns in the four acute liver injury models. The correlation between BDL vs. Sham control and LR vs. Muscle resection (MR) control was the strongest (R = 0.87). Correlations between ConA vs. NS control and the others were weak. CCl4 vs. Oil control was negatively correlated with the others (**Figure 3B**). Correlations between metabolites in each group were showed in **Figure 3, C-F**. Correlations with an absolute value over 0.75 in CCl4, ConA, BDL, and LR were 108, 75, 328, and 259 respectively. Correlations between metabolites in BDL and LR groups were much stronger than those in CCl4 and ConA groups. In both BDL and LR groups, saturated fatty acid (SFA) was negatively correlated to saturated fatty acid (UFA). Furthermore, the ratio of SFA to UFA in these two groups was dramatically increased (**Figure 3G**).

### Potential crosstalk between liver transcriptome and serum metabolome

**Figure 4:**
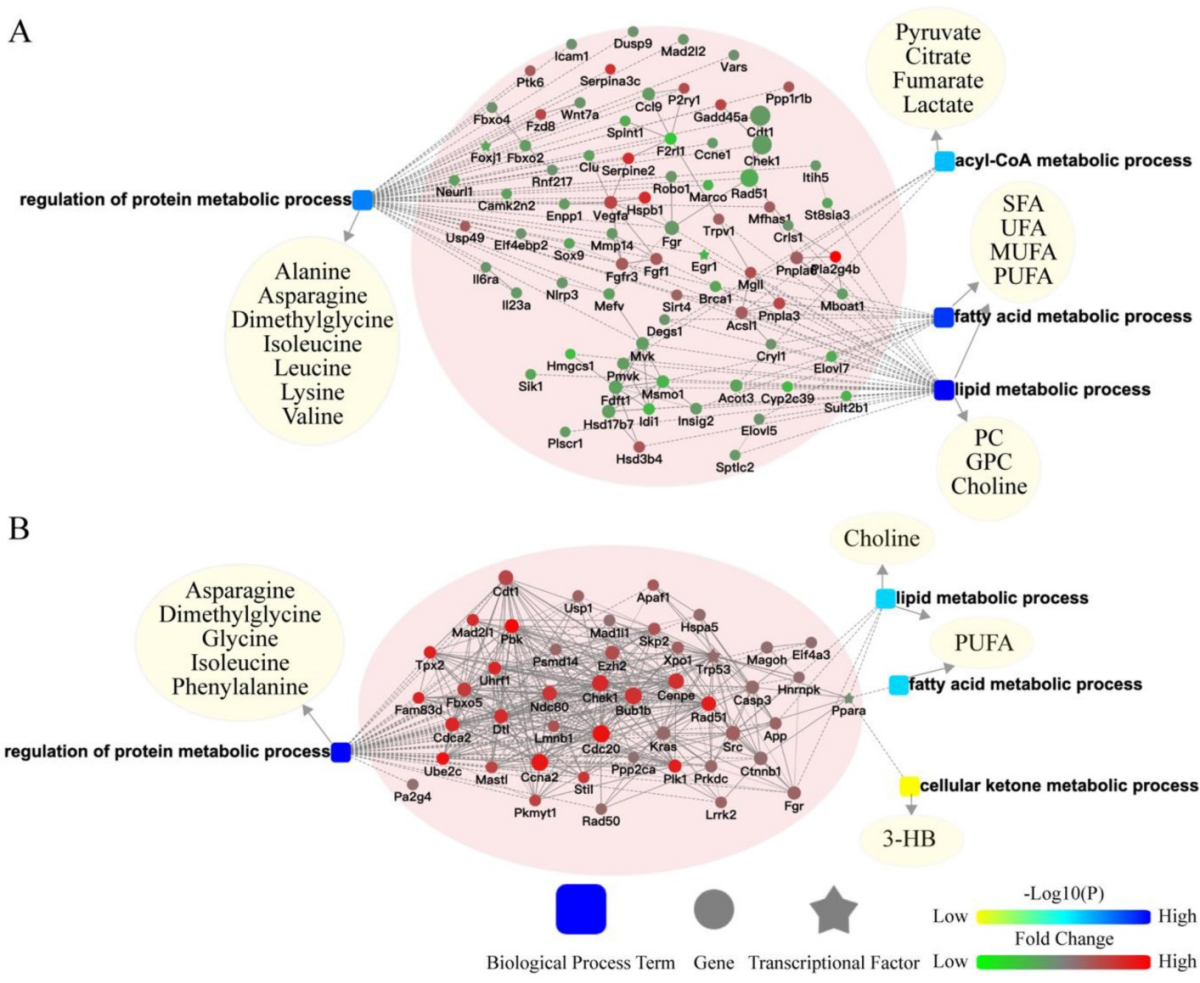
** Potential crosstalk between liver transcriptome and serum metabolome.** The biological process based-network for genes changed in liver tissues and metabolites changed in serum revealed the effect of primary acute liver injury on the secondary changes in serum metabolites. (A) Potential liver genes-serum metabolites crosstalk network for BDL group. (B) Potential liver genes-serum metabolites crosstalk network for LR group. (3-HB, 3-hydroxybutytrate. SFA, saturated fatty acid. UFA, unsaturated fatty acid. MUFA, monounsaturated fatty acid. PUFA, polyunsaturated fatty acid. GPC, glycerophosphorylcholine. PC, phosphorylcholine. TMAO, trimetlylamine oxide. NAG, N-acetylated glycoproteins. OAG, O-acetylated glycoproteins.)

To better understand the potential crosstalk between liver and extra-liver organs, two surgical models (BDL and LR) were chosen for further analyses. We built a network of liver transcriptomic changes and serum metabolomic changes based on biological processes to explore the mechanism of metabolomic change. In the liver of BDL model, changed genes related to metabolism were enriched in regulation of protein metabolic process, acyl-CoA metabolic process, fatty acid metabolic process, and lipid metabolic process (**Figure 4A**). In the liver of LR model, changed genes related to metabolism were enriched in the regulation of protein metabolic processes, fatty acid metabolic processes, lipid metabolic processes, and cellular ketone metabolic processes (**Figure 4B**). Serum differential metabolites were classified in these biological processes. The genes-biological processes-metabolites network showed the pattern in which the hepatic transcriptome may regulate the serum metabolome after acute liver injury.

### The potential mechanism of pulmonary damage after BDL-induced acute liver injury

**Figure 5:**
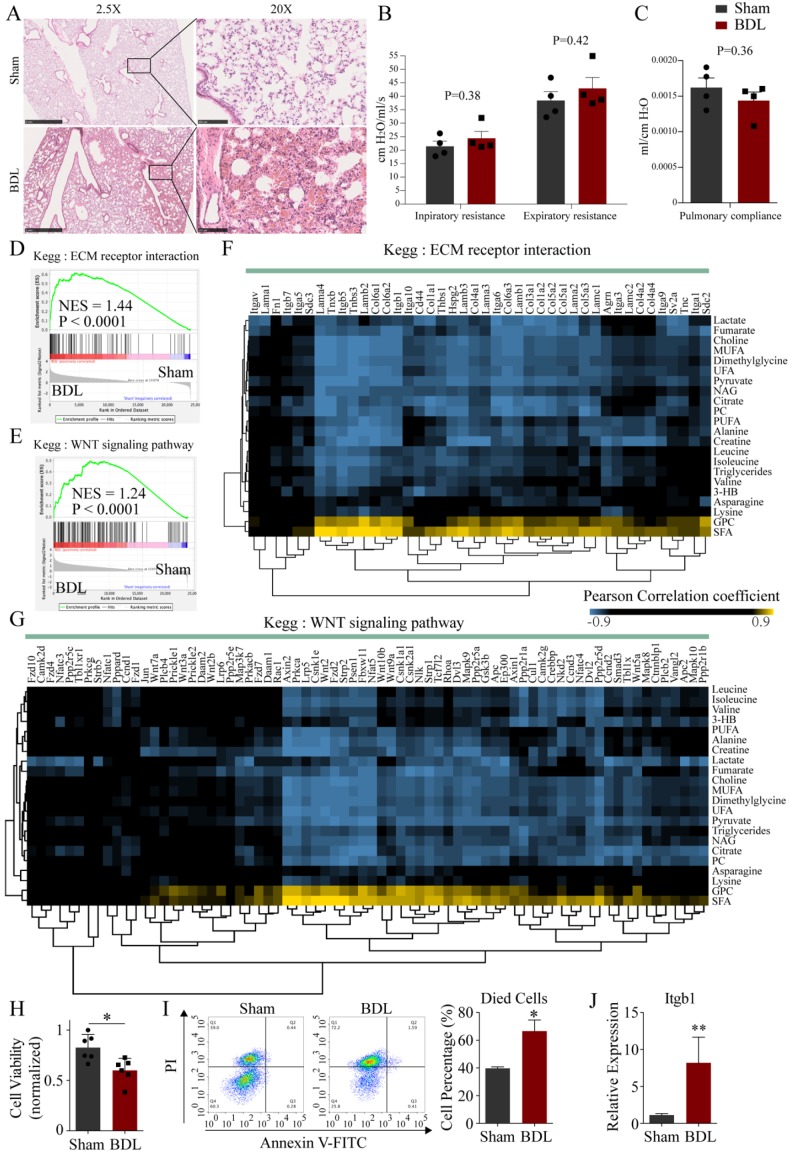
** The potential mechanism of pulmonary damage after BDL-induced acute liver injury.** (A) Representative H&E staining of lung tissue sections from the BDL mice. Scar bar on the left, 1 mm. Scar bar on the right, 100 μm. (B) Inpiratory and expiratory resistance in BDL mice. (C) Pulmonary compliance in BDL mice. GSEA for pulmonary transcriptome showed ECM receptor interaction (D) and WNT signaling pathway (E) were upregulated. Correlation heatmaps showed correlations between metabolites changed in BDL serum and genes enriched in ECM receptor interaction (F) and WNT signaling pathway (G) indicated the potential crosstalk between serum metabolites and pulmonary transcriptome after BDL-induced liver injury. Correlations with an absolute value greater than 0.9 were colored in heatmap. (H) Viability of MLE-12 cells treated with serum derived from BDL and Sham mice. (I) Apoptotic cells detection by flow cytometry. Left, representative flow cytometry images. Right, statistic histogram images of died cells. (J) Itgb1 expression of MLE-12 cells treated with serum derived from BDL and Sham mice detected by QPCR. (3-HB, 3-hydroxybutytrate. SFA, saturated fatty acid. UFA, unsaturated fatty acid. MUFA, monounsaturated fatty acid. PUFA, polyunsaturated fatty acid. GPC, glycerophosphorylcholine. PC, phosphorylcholine. TMAO, trimetlylamine oxide. NAG, N-acetylated glycoproteins.)

H&E staining of lung tissues of mice with BDL showed local pulmonary hemorrhage and alveolar wall thickening (**Figure 5A**). We tested airway function in BDL mice. The inpiratory and expiratory resistance was slightly increased in BDL with no significance (**Figure 5B**). The pulmonary compliance was slightly decreased in BDL with no significance (**Figure 5C**). These results suggested The lung developed mild histological damage after BDL, but did not impair the airway function. Next, we analyzed the lung transcriptome in BDL mice. Principal components analysis (PCA) showed that the transcriptomic profile of the lung tissue in BDL was distinctly different from that in control (**Supplementary Figure 9A**). Gene set enrichment analysis (GSEA) was performed to explore the intrinsic mechanism of pulmonary histological changes after BDL. There were 36 gene sets upregulated and no gene set downregulated significantly after BDL. Among the 36 gene sets, ECM receptor interaction and WNT signaling pathway were reported to related to lung development and injury 26-28. GSEA plots of ECM receptor interaction (NES=1.44, P<0.0001) and WNT signaling pathway (NES=1.24, P<0.0001) were showed (**Figure 5D, E**). Pearson correlation between serum differential metabolites and genes enriched in these two pathways was performed (**Figure 5F, G**). SFA was positively correlated while UFA was negatively correlated to genes in these two pathways.

To further demonstrate the effects of BDL-induced acute liver injury on the lung, we treated the MLE-12 cells (type II alveolar epithelial cells) with serum from BDL and Sham mice. In result, the viability of MLE-12 cells treated with serum from BDL mice was significantly decreased compared with that from Sham mice (**Figure 5H**). The percentage of died cells in BDL serum-treated group was significantly increased compared with that in Sham serum-treated group by flow cytometry (**Figure 5I**). QPCR analysis revealed that Itgb1 was significantly up-regulated after BDL serum treatment, which was consistent with the results of RNA-seq (**Figure 5J**).

### The effects of BDL and LR on the renal and brain transcriptomics

**Figure 6:**
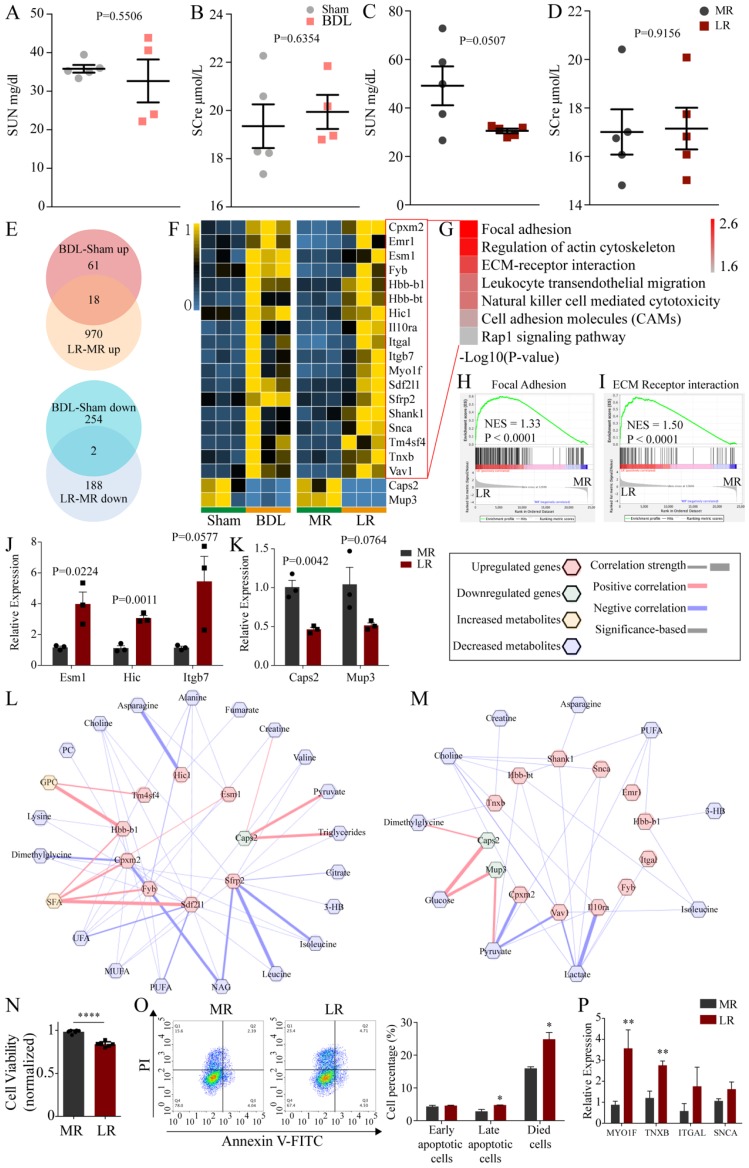
** The effects of BDL and LR on the renal transcriptomics. (A-D) Renal function testing through serum biochemical testing.** (A) SUN in Sham and BDL mice. (B) SCre in Sham and BDL mice. (C) SUN in MR and LR mice. (D) SCre in MR and LR mice. (E) Venn plots for up-regulated and down-regulated genes in renal transcriptome of BDL and LR models. (F) Heatmaps for co-changed genes in renal transcriptome of BDL and LR models. (G) Kegg pathway enrichment for co-upregulated genes in renal transcriptome of BDL and LR models. GSEA plots in renal transcriptome of LR showed focal adhesion (H) and ECM receptor interaction (I) were upregulated. (J) Quantitative-PCR validation of expression changes of Esm1, Hic and Itgb7 in LR models. (K) Quantitative-PCR validation of expression changes of Caps2 and Mup3 in LR models. (L) Correlation between co-changed genes in renal transcriptome and serum changed metabolites in BDL models. (M) Correlation between co-changed genes in renal transcriptome and serum changed metabolites in LR models. Pearson coefficient with an absolute value greater than 0.95 was displayed. Up-regulated genes were shown as red terms and down-regulated genes were shown as green terms. Increased metabolites were shown as yellow terms and decreased metabolites were shown as blue terms. Each correlation was displayed as a line across genes and metabolites. The width of the line indicated the correlation strength. Positive correlation was shown as red lines and negative correlation was shown as blue lines. (N) Viability of human podocytes treated with serum derived from LR and MR mice. (O) Apoptotic cells detection by flow cytometry. Left, representative flow cytometry images. Right, statistic histogram images of early apoptotic cells, late apoptotic cells, and died cells. (P) MYO1F, TNXB, ITGAL, and SNCA expression of human podocytes treated with serum derived from LR and MR mice detected by QPCR. (SUN, serum urea nitrogen. SCre, serum creatinine. CCl4, carbon tetrachloride. NS, normal saline. ConA, concanavalin A. BDL, bile duct ligation. MR, muscle resection. LR, liver resection. 3-HB, 3-hydroxybutytrate. SFA, saturated fatty acid. UFA, unsaturated fatty acid. MUFA, monounsaturated fatty acid. PUFA, polyunsaturated fatty acid. GPC, glycerophosphorylcholine. PC, phosphorylcholine. TMAO, trimetlylamine oxide. NAG, N-acetylated glycoproteins. OAG, O-acetylated glycoproteins.)

H&E staining showed no significant changes in kidney tissue (**Figure 1E**). Then, we tested renal function (Serum urea nitrogen, SUN; Serum creatinine, SCre) in BDL and LR group. The SUN of LR mice was reduced with no significance (P=0.0507) (Figure 6C). No other abnormalities were found in other indicators of renal function (**Figure 6A, B, D**). Next, we analyzed the kidney transcriptome. PCA showed that the transcriptomic profile of the kidney in these two models was distinctly different from that in the respective control (**Supplementary Figure 9B, C**). Venn plots showed the co-changed genes of renal transcriptome in two models (**Figure 6E**) and these genes were showed in heatmap (**Figure 6F**). Co-upregulated genes are significantly enriched in seven pathways including focal adhesion, regulation of actin cytoskeleton, ECM-receptor interaction, leukocyte transendothelial migration, natural killer cell mediated cytotoxicity, cell adhesion molecules (CAMs), rap1 signaling pathway (**Figure 6G**). Gene Set Enrichment Analysis (GSEA) showed that focal adhesion (NES=1.33, P<00001) and ECM receptor interaction (NES=1.50, P<0.0001) were upregulated significantly in the LR group (**Figure 6H, I**). We performed Quantitative Real-time PCR (Q-PCR) to validate the change of Esm1, Hic and Itgb7 expression in LR models (**Figure 6J**). The predictive Protein-Protein Interaction (PPI) of co-upregulated genes was showed in **Supplementary Figure 10**. There were two genes co-downregulated in these two models, Caps2 and Mup3, which encode calcyphosin-2 and major urinary proteins 3 respectively. We performed Quantitative Real-time PCR (Q-PCR) to validate the change of Caps2 and Mup3 expression in LR models (**Figure 6K**). Potential crosstalk networks between serum metabolites and co-changed genes in BDL and LR were depicted based on Pearson correlation with an absolute value greater than 0.95 (**Figure 6L, M**).

To further demonstrate the effects of LR-induced acute liver injury on the kidney, we treated the human podocytes (HPs) with serum from LR and MR mice. In result, the viability of HPs treated with serum from LR mice was significantly decreased compared with that from MR mice (Figure 6N). The percentage of late apoptotic cells and died cells in LR serum-treated group was significantly increased compared with that in MR serum-treated group by flow cytometry (Figure 6O). QPCR analysis revealed that MYO1F and TNXB were significantly up-regulated after LR serum treatment, which was consistent with the results of RNA-seq (Figure 6P).

**Figure 7:**
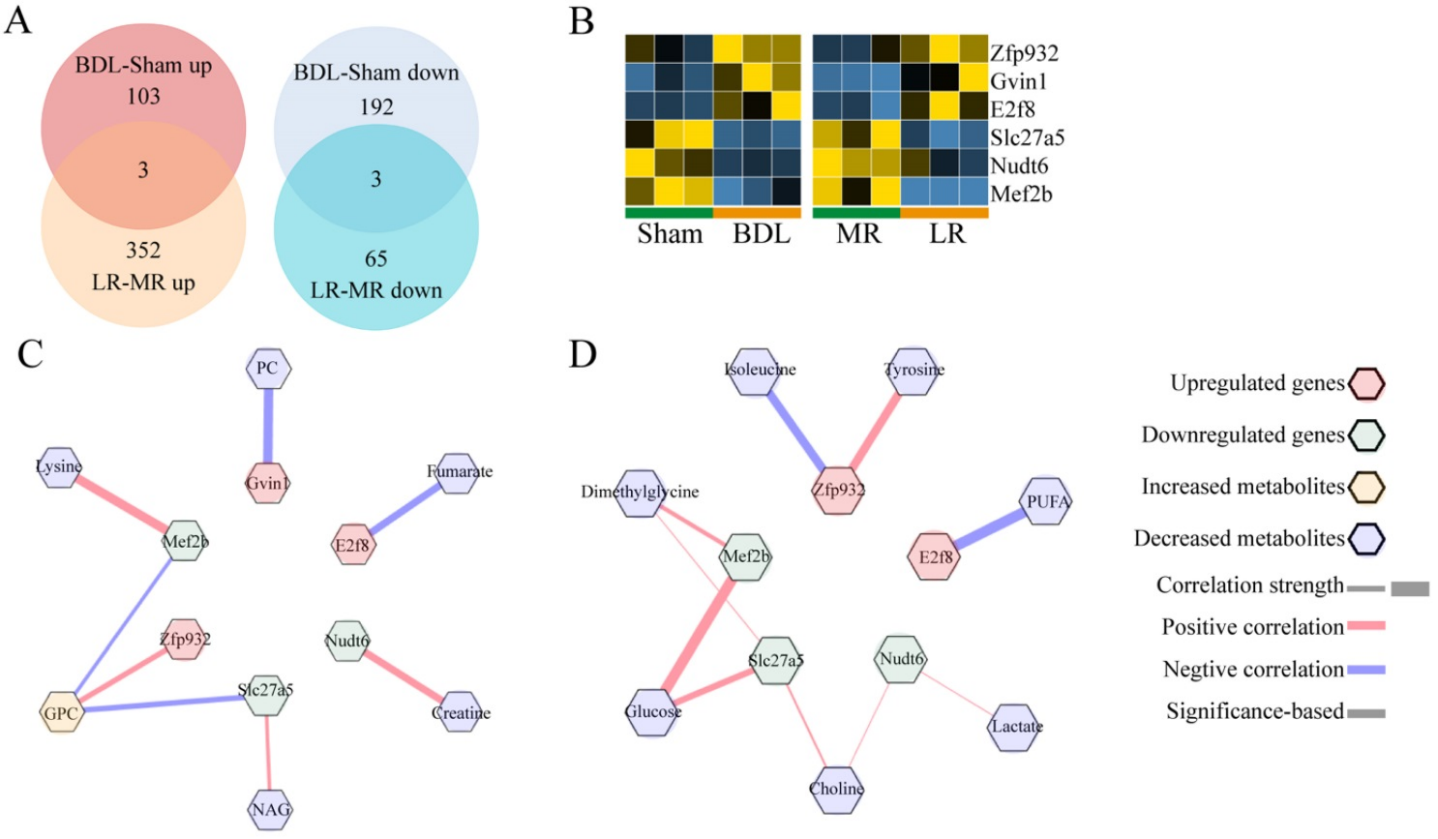
** The effects of BDL and LR on the brain transcriptomics.** (A) Venn plots for changed genes in brain transcriptome of BDL and LR models. (B) Heatmaps for co-changed genes in renal transcriptome of BDL and LR models. (C) Correlation between co-changed genes in brain transcriptome and changed serum metabolites in BDL models. (D) Correlation between co-changed genes in brain transcriptome and changed serum metabolites in LR models. Pearson coefficient with an absolute value greater than 0.95 was displayed. Up-regulated genes were shown as red terms and down-regulated genes were shown as green terms. Increased metabolites were shown as yellow terms and decreased metabolites were shown as blue terms. Each correlation was displayed as a line across genes and metabolites. The width of the line indicated the correlation strength. Positive correlation was shown as red lines and negative correlation was shown as blue lines.

We also analyzed the brain transcriptome to find the potential mechanism of HE. PCA analyses showed that the transcriptomic profile of the kidney in these two models was distinctly different from that in the respective control (**Supplementary Figure 9D, E**). There were only 3 genes co-upregulated and 3 genes co-downregulated significantly as indicated (**Figure 7A, B**). Potential crosstalk networks between serum metabolites and co-changed genes in BDL and LR were depicted based on Pearson correlation with an absolute value greater than 0.95 (**Figure 7C, D**).

## Discussion

CCl4, ConA, BDL, and LR-induced acute liver injury models are widely used in the research field of liver diseases. However, the systematic changes of multiple organs in these models and the potential crosstalk between liver and extra-liver organs has not been carefully investigated. In the present study, the changes of morphology, serum metabolome, and transcriptome of multiple organs were performed and potential crosstalk between liver and extra-liver organs mediated by serum was analyzed.

Intriguingly, significant changes of transcriptome were observed in multiple extra-liver organs despite dramatic morphological damage only occurred in lung tissues of ConA/BDL group and spleen tissues in ConA group. Although morphological changes were not obvious in other organs, the transcriptional changes are likely to initiate tissues damage and eventually lead to the organs dysfunction in the long run.

Because CCl4 and ConA enter into the circulatory system and might directly act on the extra-liver organs, it is difficult to distinguish between primary effects and secondary effects induced by liver injury. BDL and LR models were chosen for deeper analyses of potential crosstalk between liver and extra-liver organs in liver injury. The effects of these two surgeries on serum metabolome were similar. Most of serum metabolites in BDL and LR were decreased, which indicated that the mice in BDL and LR group were in a state of energy exhaustion and metabolic disorder. However, transcriptomic changes in BDL and LR liver were distinguished, which suggested that the way that different causes of liver injury drove different patterns of change in serum metabolites.

Upon BDL, dramatic morphological damage was observed in lung tissues, which was consistent with previous studies 20, 21. ECM receptor interaction and WNT signaling pathway were upregulated in lung tissues of BDL group. Upon injury or with aging, dynamic changes within the ECM are associated with several chronic lung diseases 26. Through pathway enrichment analysis to a meta-analyzed GWAS, Gharib et al. noted enrichment of ECM processes specifically in the airflow obstruction study 27. WNT signaling pathway were reported to regulate the asthmatic airway remodeling, which induced by pulmonary chronic inflammation 28. Based on joint analysis of serum metabolome and pulmonary transcriptome, we found that SFA was positively, while UFA, MUFA, and PUFA were negatively correlated to genes in the above two pathways. The increased SFA/UFA ratio might play an important role in pulmonary damage upon liver injury. Our data showed that serum derived from BDL-induced acute liver injury mice impaired the viability of alveolar epithelial cells *in vitro*. This suggests that the change of substances in serum after BDL-induced liver injury damages the lung and mediates the crosstalk between lung and liver.

HRS is an important complication of acute liver injury. In our study, despite no renal histological changes were observed in BDL and LR models, the renal transcriptomic changes were significant in these two models. Among the 18 genes co-upregulated in BDL and LR models, Fyb, Sfrp2, Hic1, Itgb7, and Esm1 were reported to participate in renal development or diseases 29-34. The 18 genes upregulated simultaneously in these two models were enriched in focal adhesion related pathways. Focal adhesion plays an important role in protecting kidney from injury through podocytes. Loss of podocytes adhesion is a hallmark of glomerular disease progression. Rap1 was reported as an important mediator for integrin activation and rap1 reduction in podocytes led to severe renal disease 35-37. At the early stages of cholestasis and ALF model, changes in podocyte behavior might be crucial. On the other hand, leukocyte transendothelial migration and natural killer cell-mediated cytotoxicity might be related with the renal injury. As reported, Itgb7 was the marker of renal inflammation 32, 34. Though without histological change, kidney might suffer from inflammation at the early stage of acute liver injury. The accumulation of chronic and mild inflammation will eventually lead to renal damages, lesions and even dysfunction. As indicated in **Figure 6B**, Caps2 and Mup3 were downregulated in kidney after BDL and LR. It is worth to further study whether the expression of these two proteins are decreased in kidney after liver injury. Therefore, Caps2 and Mup3 might be the potential biomarkers in HRS, which contribute to early diagnosis of HRS. In BDL, metabolites highly correlated with the above co-changed genes included SFA, GPC, pyruvate, and amino acids. Moreover, metabolites highly correlated with the above co-changed genes were glucose, pyruvate, and lactate in LR, which suggested the important role of glycolysis in development of HRS induced by LR. Our data also showed that serum derived from LR-induced acute liver injury mice impaired the viability of human podocytes *in vitro*. This suggests that the change of substances in serum after LR-induced liver injury damages the kidney and mediates the crosstalk between kidney and liver.

HE is another vital complication of acute liver injury. Among the genes changed simultaneously in brains of BDL and LR models, E2f8 is a transcriptional factor indispensable for angiogenesis, lymphangiogenesis, embryonic development 38, and differentiation of neuroblasts 39. Based on correlation between genes and metabolites, E2f8 might be regulated by fumarate and PUFA respectively in BDL and LR. It is worth to further study whether E2f8 plays an important role in the development of HE and how the E2f8 in brain is regulated upon acute liver injury.

In summary, dramatic transcriptomic changes of extra-liver organs occurred in the four acute liver injury models commonly used in the basic and translational research of liver diseases. The influence of extra-liver organs on the pathogenesis of liver injury should be strictly considered in the future research. Crosstalk networks between liver and extra-liver organs mediated by serum metabolites exist during the development of liver injury.

## Materials and Methods

### Animals and grouping

All the protocols in this study were approved by the Guide for the Care and Use of Laboratory Animals (National Institutes of Health, Publication No. 85-23, Revised) and was carried out under the supervision of the Fudan University Institutional Animal Care and Use Committee. All experiments were performed on age-matched male mice. C57BL/6J mice were obtained from Shanghai Slake Laboratory Animal co. LTD. Mice were divide into eight groups-Oil, CCl4, NS, ConA, Sham, BDL, MR, and LR. Oil is the control group of CCl4. NS is the control group of ConA. Sham is the control group of BDL. MR is the control group of LR.

Six to eight-week-old male mice were chosen for all experiments. Mice in CCl4 group were injected intraperitoneally with 500 µl/kg CCl4 dissolving in olive oil. Mice in Oil group were injected intraperitoneally with the same volume of olive oil. Mice in ConA group were injected with five mg/kg ConA dissolving in saline. Mice in NS group were injected with the same volume of saline. Mice in Sham group were just performed abdominal incision. Mice in BDL group were suffered common bile duct ligation. Mice in MR group were cut off a piece of abdominal muscles. Mice in LR group were suffered >80% liver resection.

### Chemicals and reagents

Reagents used for this study were as follows: CCl4 (Sinopharm), ConA (Sigma), Trizol (Invitrogen), AMV Reverse Transcription System (TOYOBO), and SYBR Green PCR Master Mix (Vazyme).

### NMR Serum metabolome

Blood samples were collected immediately after sacrificed the mice and allowed to stand on ice for two hours. Serum samples were obtained by centrifuging (4 ℃, 3000 rpm, 5 min) the blood samples. Serum samples were prepared by mixing 200 μL of serum with 400 μL of phosphate buffer (45 mM, pH 7.4) containing 10% D2O. After vortexing and 10 min of centrifugation (14489 g, 4 °C), 550 μL of supernatant was transferred to a 5 mm NMR tube for each sample, followed by NMR analysis described previously 40.

### RNA sequencing

RNA was extracted from fresh tissues or cells using the Trizol and the cDNA libraries were prepared using NGS Multiplex Oligos for Illumina (xCell BioTech Co.,Ltd) as described previously 41. Briefly, the mRNA was separated from the total RNA, and then fragmented and reversed to cDNA. After end repaired, cDNA was barcoded with multiplex adapters and amplified a cDNA library. The cDNA libraries were purified with AmpureXP beads, and samples were quantified by Qubit (Invitrogen) and then run on an Illumina Hiseq (Illumina). Our data was submitted to Sequence Read Archive (SRA) database (SRA accession: PRJNA586839) from National Center for Biotechnology Information, USA.

### Bioinformatic analysis

For NMR serum metabolome, differential metabolites were analyzed by T.Test in Microsoft Excel. PLSDA was analyzed by MetaboAnalyst 42. Correlation analysis was performed by R packages (reshape2, ggplot2).

Omicsbean online platform (www.omicsbean.com:88) was used for transcriptomic analysis (Volcano plots & PCA). Up and down-regulated genes were screened through T.Test APP in Omicsbean and merged them through Venn APP in Omicsbean. Next, these genes were picked from original data and the expression of them was normalized into 0-1 to perform heatmap. Heatmaps were made by R packages (pheatmap) on R Studio platform. Gene Set Enrichment Analysis was performed by GSEA-P 43. The visualization of Correlation was performed by Metascape in Cytoscape.

### Pulmonary function testing

Mice with BDL and Sham were performed with pulmonary function testing. Mice were anesthetized with sodium pentobarbital, performed with endotracheal intubation and placed in plethysmographic chamber. Pulmonary function was detected by a lung-function analyzing system (AniRes2005, Beijing Bestlab High-Tech Company Limited, Beijing, China).

### Renal function testing

Mice with BDL/Sham and LR/MR were performed with renal function testing. Mice were anesthetized with 3% isoflurane and harvested blood from fundus venous plexus. Serum samples were obtained by centrifuging (4 ℃, 3000 rpm, 5 min) the blood samples and then tested by fully automatic bio analysis machine Chemray 800 (Rayto Life and Analytical Sciences Co., Ltd.).

### Histology and H&E staining

Fresh tissues were collected in 4% paraformaldehyde solution immediately after sacrificed the mice. The fixed tissues were processed for paraffin sections and subjected to H&E staining according to standard procedure.

### Cell Culturing and Treatment

MLE-12 cells were cultured in DMEM/F12 medium (Corning) supplemented with 2% fetal bovine serum (Gibco) at 37℃ and 5% CO_2_. Human podocytes were cultured in RPMI 1640 (Corning) supplemented with 10% fetal bovine serum (Gibco) at 37℃ and 5% CO_2_. MLE-12 cells were seeded in plated 24 h before treatment and were cultured in DMEM/F12 medium supplemented with 10% serum from BDL or Sham mice. Human podocytes were seeded in plated 24 h before treatment and were cultured in RPMI 1640 supplemented with 10% serum from LR or MR mice.

### Cell Viability

Cell viability was detected using Cell Counting Kit-8 (Biosharp). After treatment, medium was removed and cells were cultured in basic medium. CCK8 was added in every well (10 μl/well) and incubated 1 h in 37℃ and 5%CO_2_. OD value was detected in Microplate Reader (Bio Tek) at 450 nM and normalized for analysis.

### Apoptosis Detection by Flow Cytometry

Cells were digested by 0.25% trypsin without EDTA and harvested to be stained by Annexin V-FITC/PI Apoptosis Detection Kit (Elabscience) according to the manufacturer's protocol. Stained cells were harvested and detected by Flow Cytometer (BD Celesta, Beckman). Data was analyzed by FlowJo 10.4.

### Quantitative Real-time PCR

RNA was extracted from specific tissues using Trizol. cDNA was obtained by reverse transcription of RNA using the AMV Reverse Transcription System (TOYOBO). Quantitative RT-PCR was performed using SYBR Green PCR Master Mix (Vazyme) with 36B4 as an internal control. The nucleotide primer sequences were documented in the Supplementary Table 1.

## Supplementary Material

Supplementary figures and table.
