# Supplementary material for: Potential Crosstalk between Liver and Extra-liver Organs in Mouse Models of Acute Liver Injury
Source: Int J Biol Sci. 2020 Feb 10;16(7):1166–79. doi: 10.7150/ijbs.41293 (PMC7053327; doi:10.7150/ijbs.41293)
Supplement: Supplementary file 1 — Supplementary figures and table. [file ijbsv16p1166s1.pdf]

Supplementary Figure 1: Large view of liver and heart section images (Figure 1A, B).

Supplementary Figure 2: Large view of lung and kidney section images (Figure 1C, D).

Supplementary Figure 3: Large view of spleen and brain section images (Figure 1E, F).

Supplementary Figure 4: Volcano plots for multiple organs in the four liver injury models. Number of significantly upregulated and downregulated genes was indicated on the top of the volcano plots.

Supplementary Figure 5: Gene Ontology analysis for liver transcriptome in the four liver injury models.

Supplementary Figure 6: Pathway enrichment for genes changed in multiple organs after acute liver injury. According to the P-value, top 5 pathways were showed.

Supplementary Figure 7: Venn plots for up- and down-regulated genes in different models of the same organ. Genes co-upregulated in three models were indicated.

Supplementary Figure 8: PLS-DA score plots for serum metabolome of the 4 acute liver injury models.

Supplementary Figure 9: PCA plots. (A) PCA plots for pulmonary transcriptome in BDL model. (B-C) PCA plots for renal transcriptome in BDL and LR models. (D-E) PCA plots for brain transcriptome in BDL and LR models.

Supplementary Figure 10: Protein-protein interaction for genes co-changed in the renal transcriptome of BDL and LR models.

Supplementary table 1: Document of primer sequence.

Supplementary Figure 1

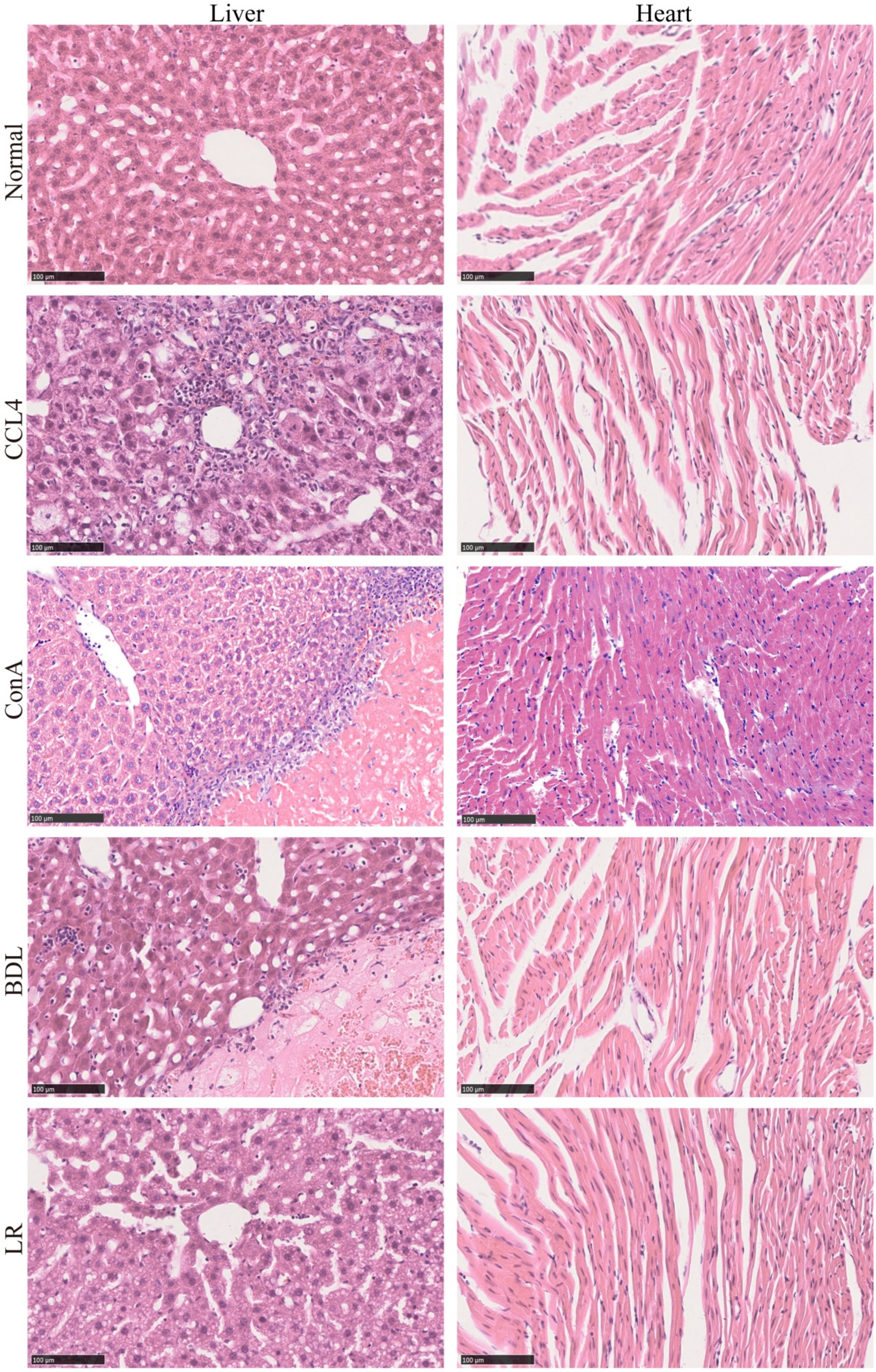

Supplementary Figure 2

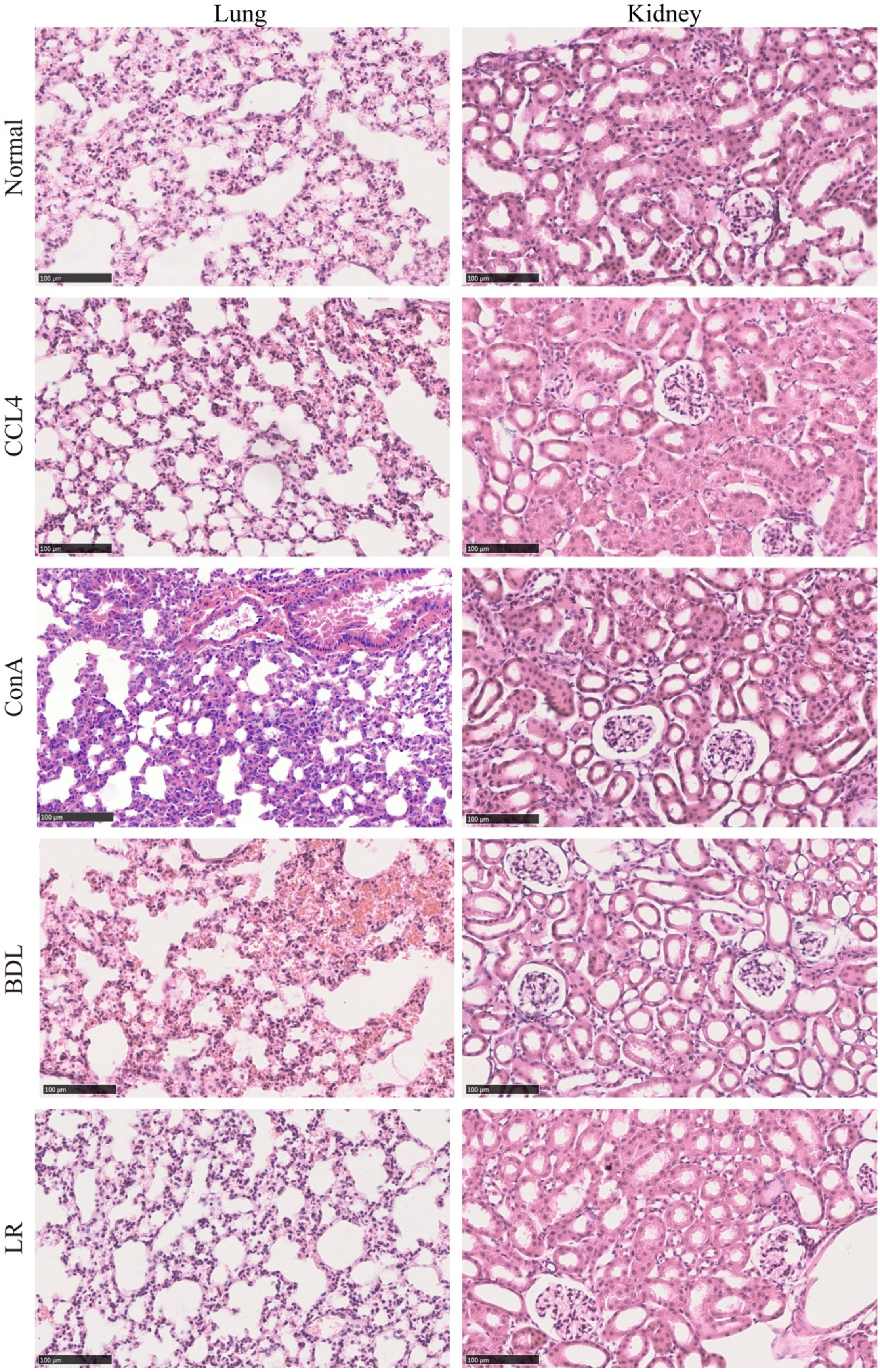

Supplementary Figure 3

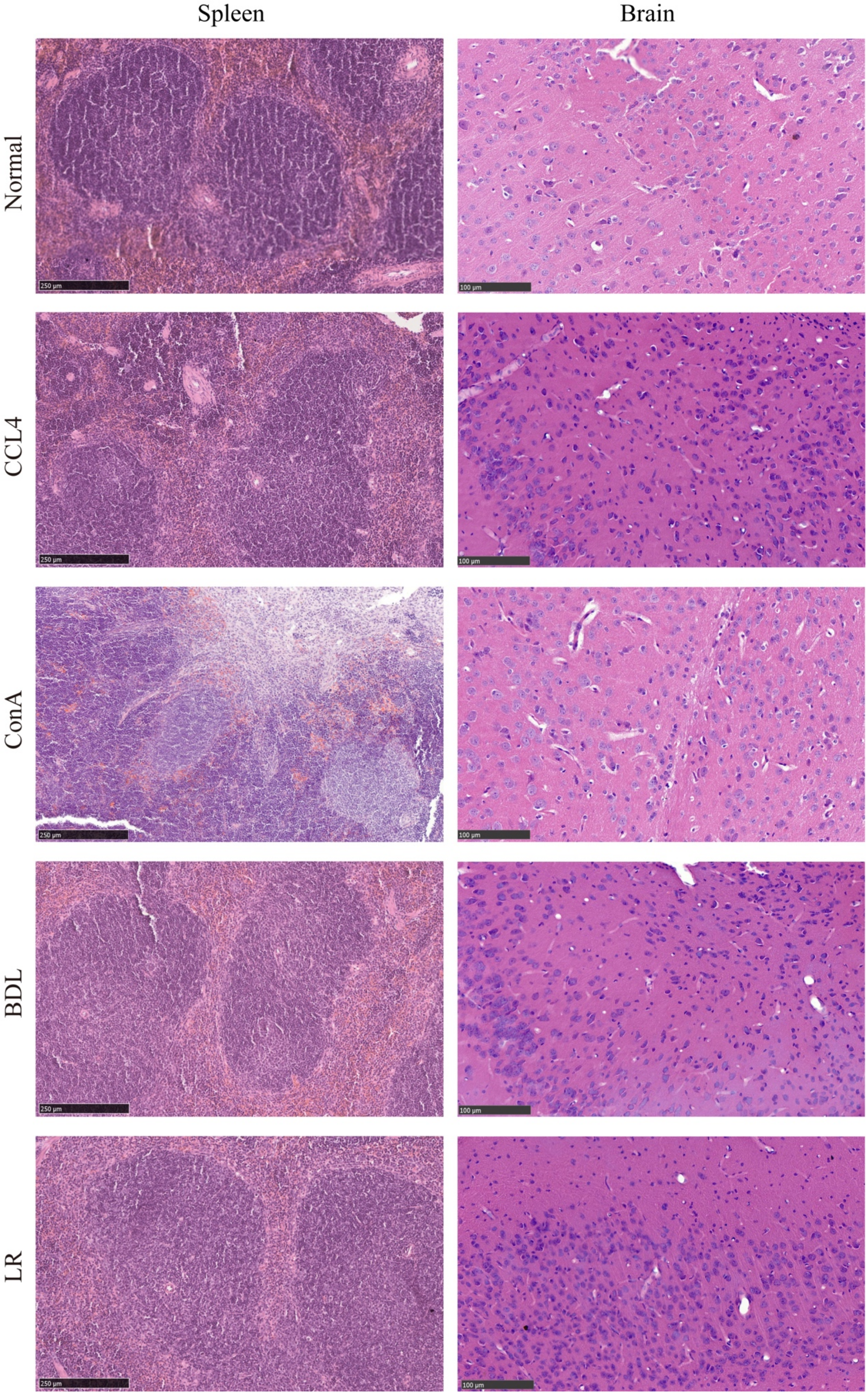

Supplementary Figure 4

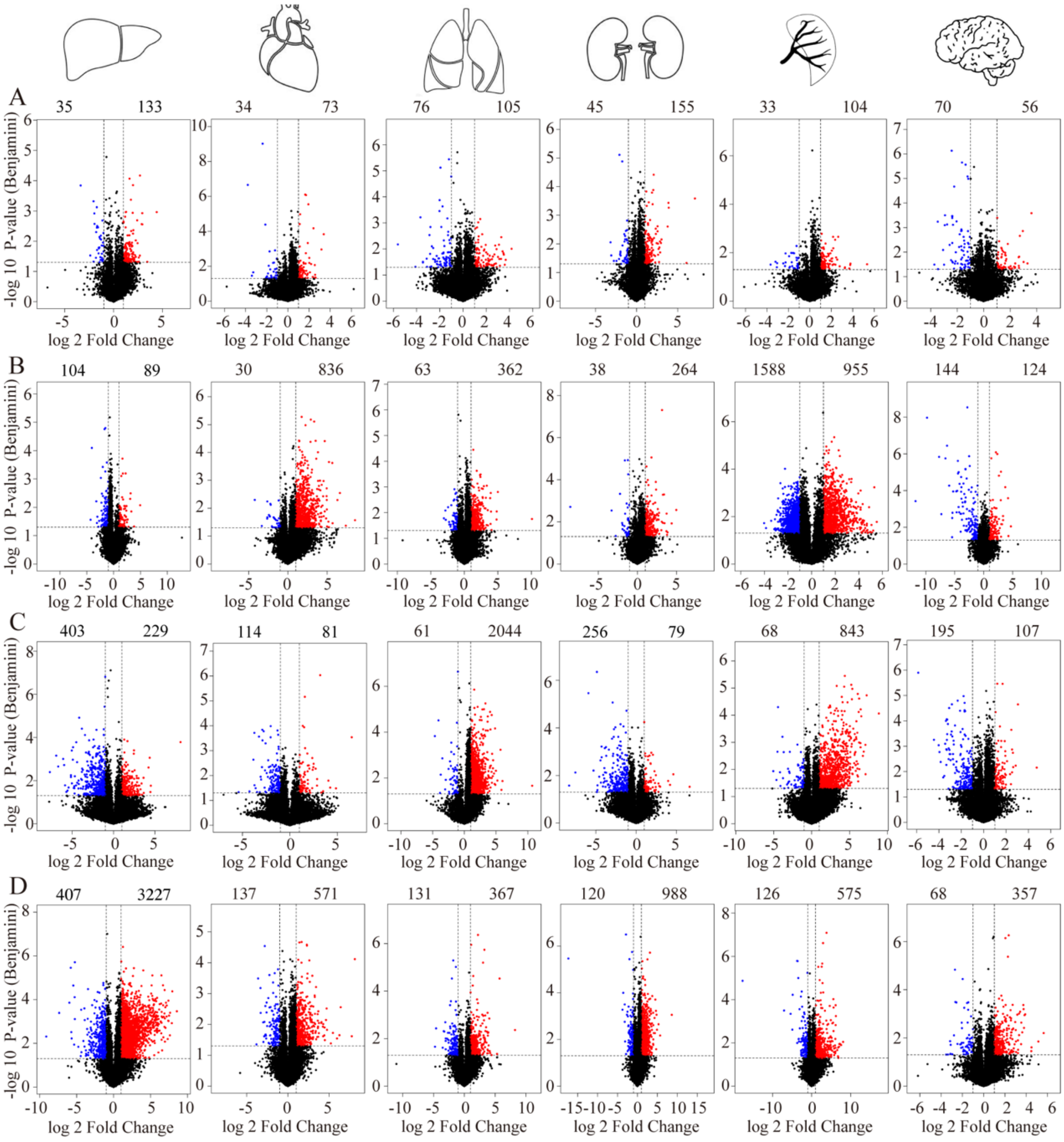

Supplementary Figure 5

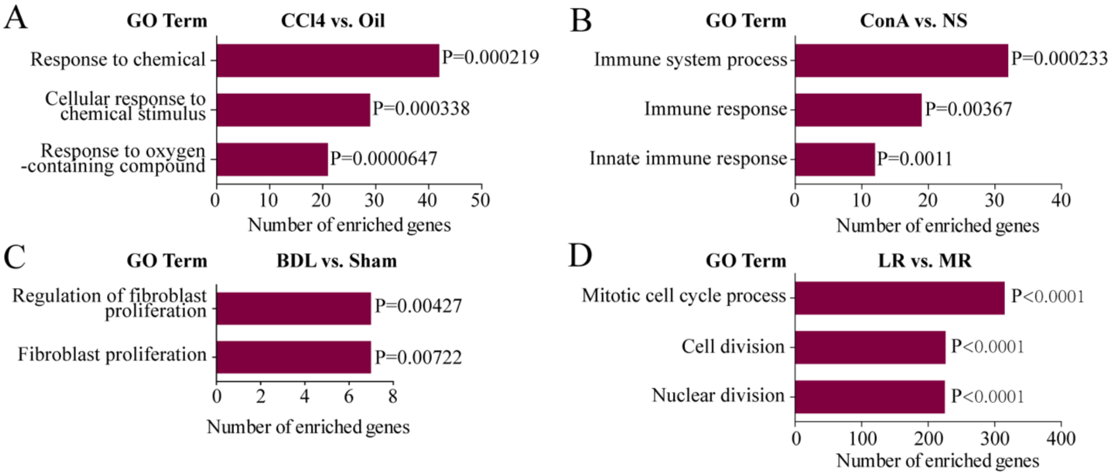

Supplementary Figure 6

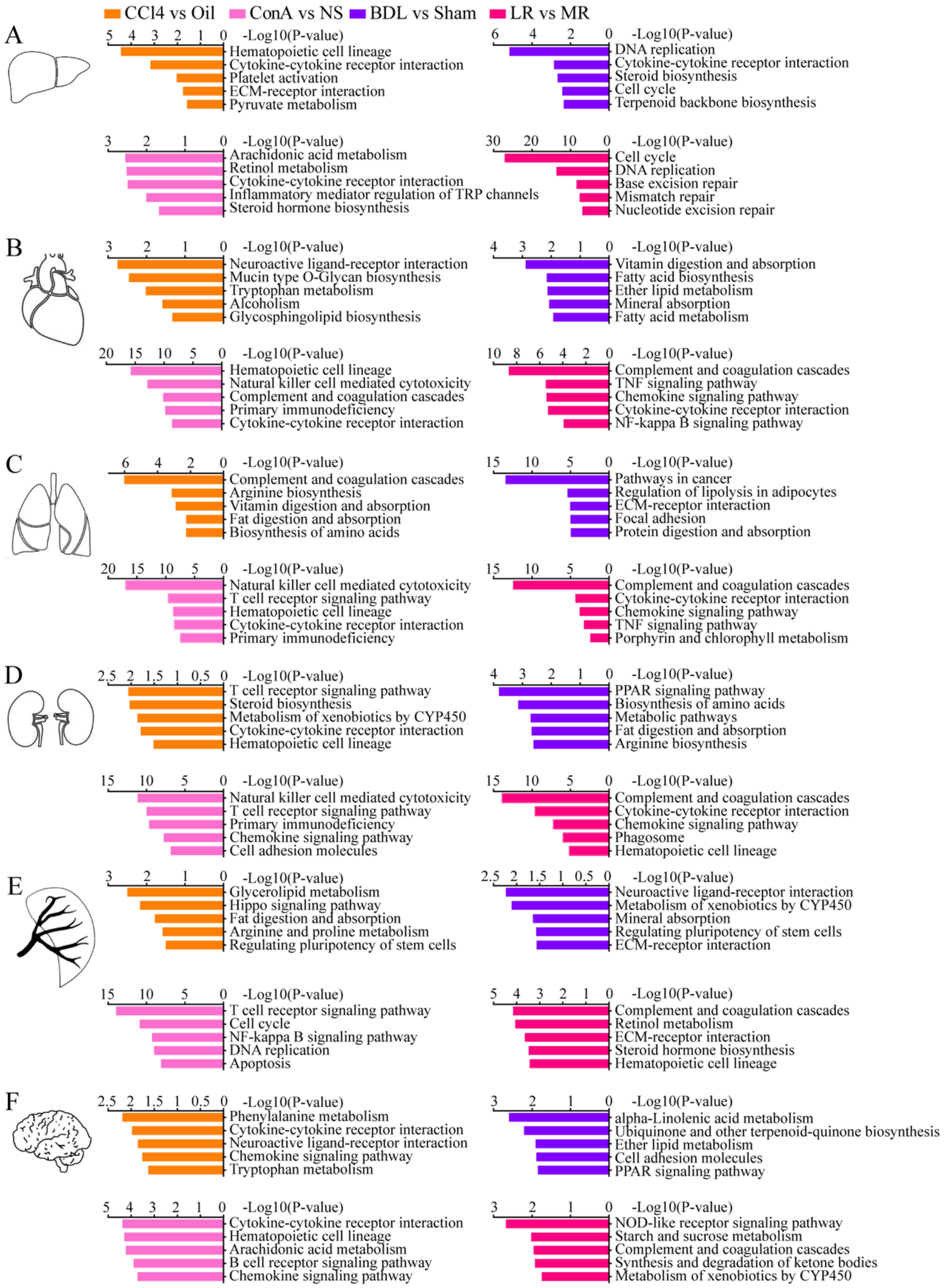

Supplementary Figure 7

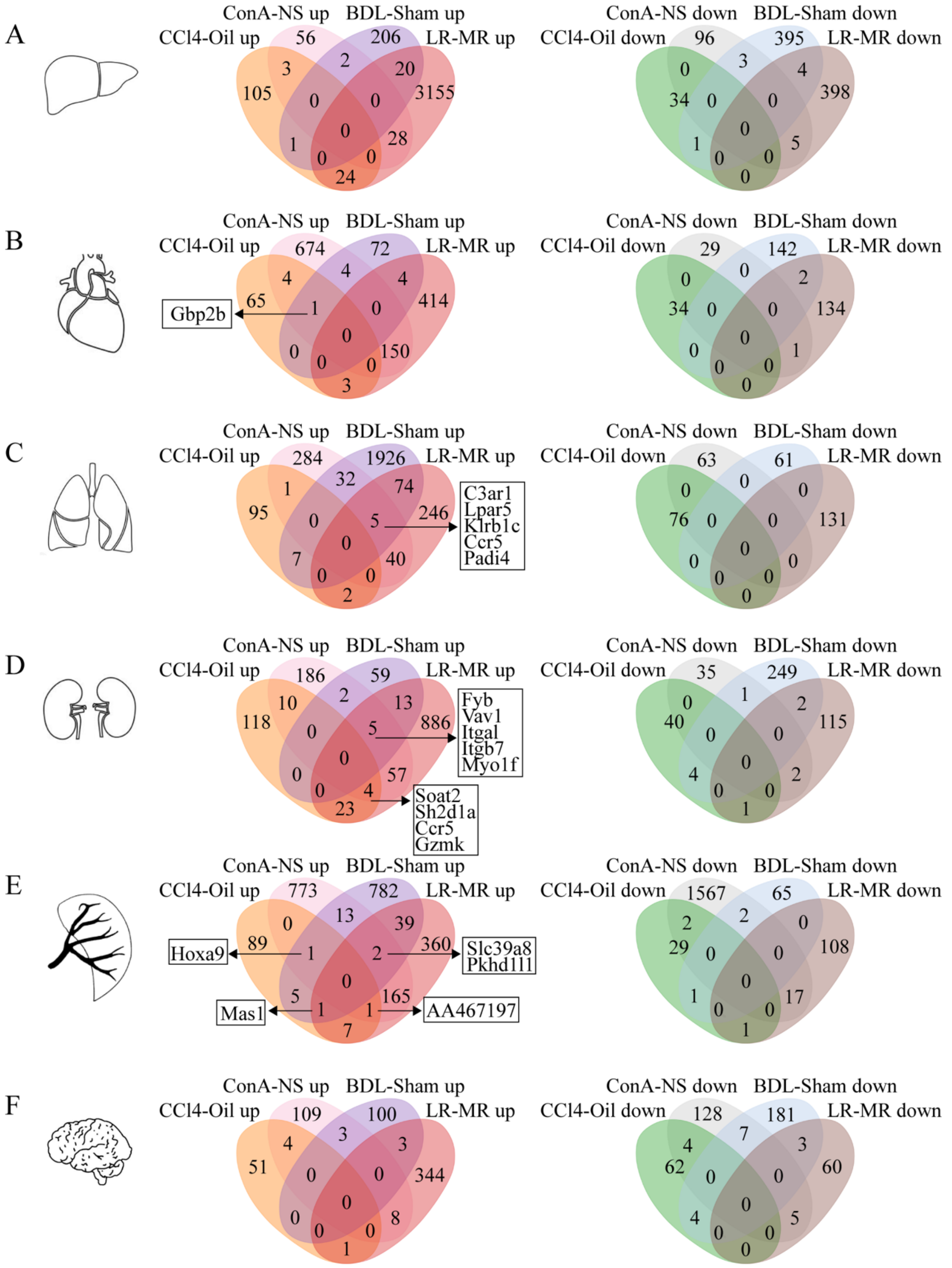

Supplementary Figure 8

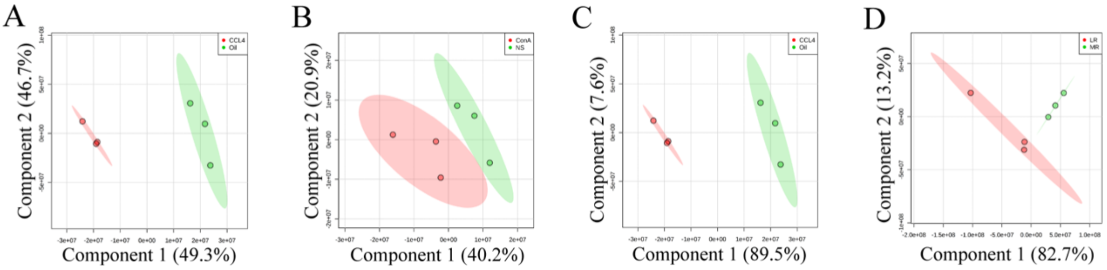

Supplementary Figure 9

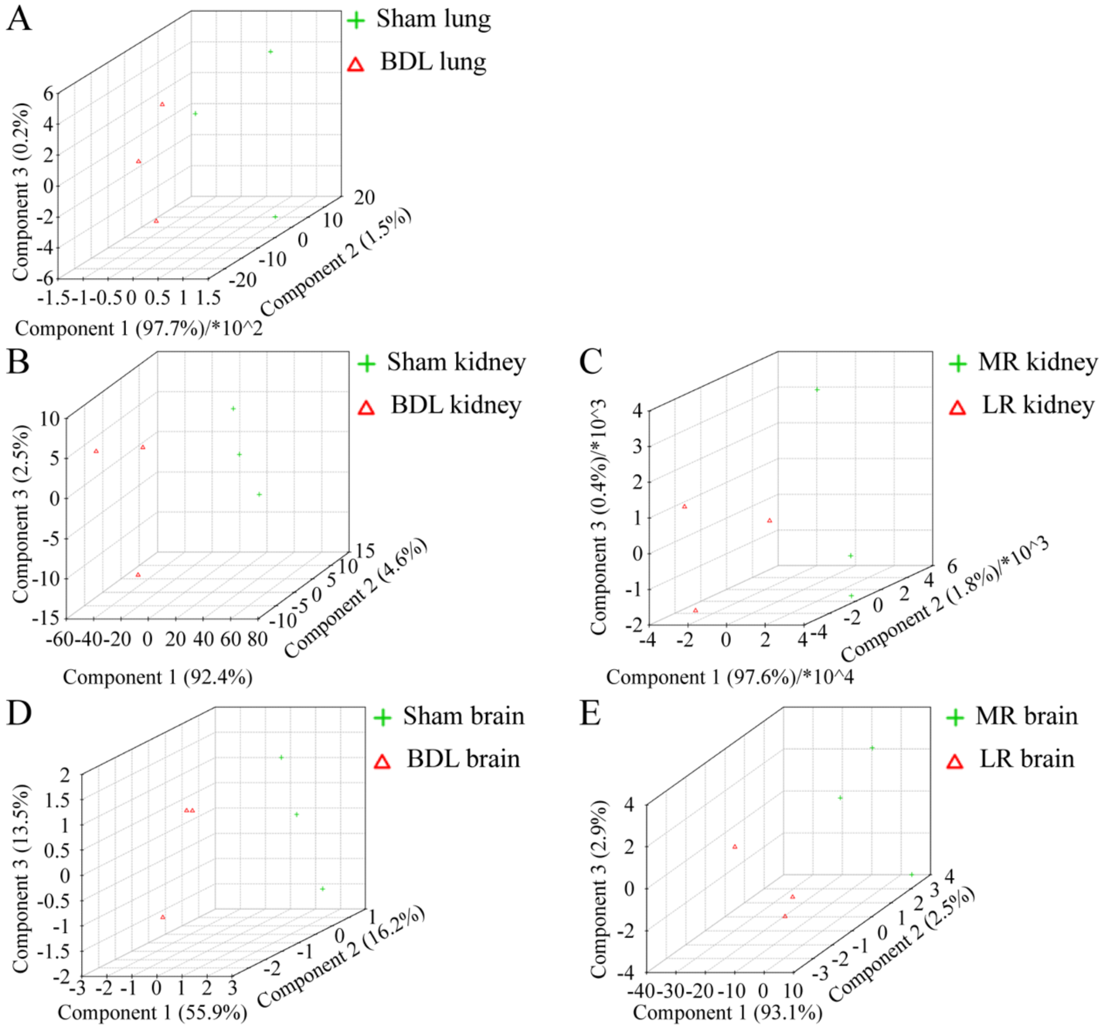

Supplementary Figure 10

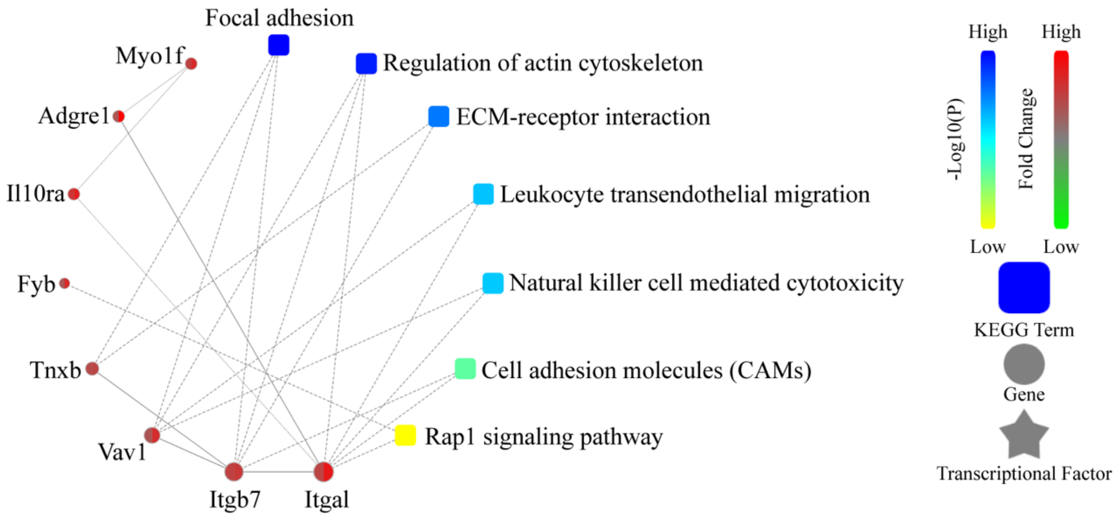

Supplementary Table 1

Name	Sequence
m-Itgb7-F	ACCTGAGCTACTCAATGAAGGA
m-Itgb7-R	CACCGTTTTGTCCACGAAGG
m-Hic1-F	GGCCATTCGAGGCAGCTAC
m-Hic1-R	AGGTTTAGCAGGTTGTCATGC
m-Esm1-F	CTGGAGCGCCAAATATGCG
m-Esm1-R	TGAGACTGTACGGTAGCAGGT
m-Caps2-F	TCTTTCTGGCTAATTCTGCAAGG
m-Caps2-R	CTTGGCGCAGTAACACTTTCT
m-Mup3-F	TTGGTTTTCTATTGCTGAAGCCT
m-Mup3-R	CCAATCGCAGTCATTTCGGTG
m-Itgb1-F	ATGCCAAATCTTGCGGAGAAT
m-Itgb1-R	TTTGCTGCGATTGGTGACATT
m-36B4-F	GGGCATCACCACGAAAATCTC
m-36B4-R	CTGCCGTTGTCAAACACCT
h-MYOF1-F	ACTACCTCAACCAATCGGACA
h-MYOF1-R	GCACTCAGAGTCTCACCAAAG
h-TNXB-F	GCCCTGCTCACTTGGACTG
h-TNXB-R	GGAGCCGTGCATTGTAGGAG
h-ITGAL-F	TGCTTATCATCATCACGGATGG
h-ITGAL-R	CTCTCCTTGGTCTGAAAATGCT
h-SNCA-F	AAGAGGGTGTTCTCTATGTAGGC
h-SNCA-R	GCTCCTCCAACATTTGTCACTT
h-36B4-F	AGCCACGCTGCTGAACAT
h-36B4-R	CAACATTGCGGACACCCT
